# Prof. S.N. Sharma: Tribute to a Teacher and Father (1934–2021)

**DOI:** 10.1055/s-0044-1800875

**Published:** 2025-01-15

**Authors:** Sandeep Sharma

**Affiliations:** 1 Aesthetique, Plastic and Cosmetic Surgery Centre, Vadodara, Gujarat, India

It has taken me a while to come around to writing this eulogy. I do not know whether it is my inherent procrastination, perhaps some denial or a subconscious desire to find space before I look back at my father in a more balanced perspective, accepting his passing away and the void in my life.

I now realize that a man with few words can say a lot. To me, he epitomized dignity in silence ( Fig. 1 ).

**Fig. 1:**
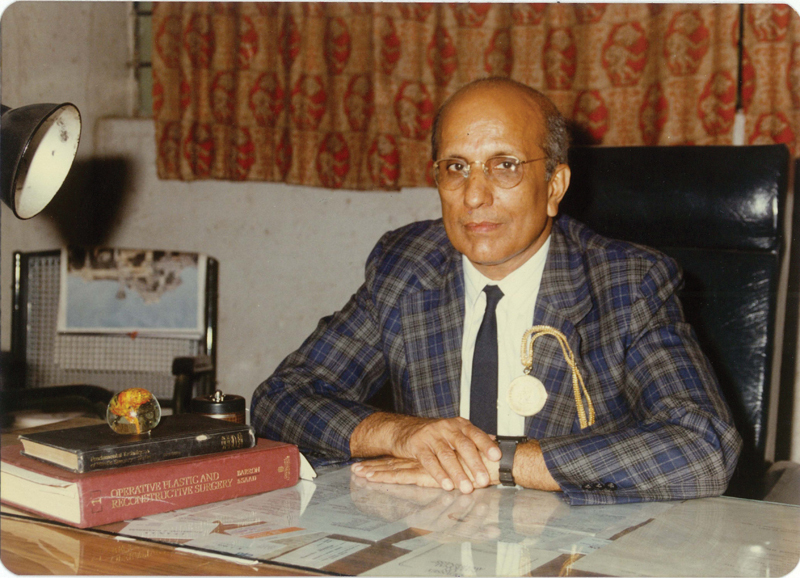
Prof. S.N. Sharma (1989).

A multifaceted personality and a past president of the Association of Plastic Surgeons of India (APSI), he was completely dedicated to the field of plastic surgery. He valued family, friends, and staff including students. He loved to travel and enjoyed photography and sports.

## Academic

**Fig. 2:**
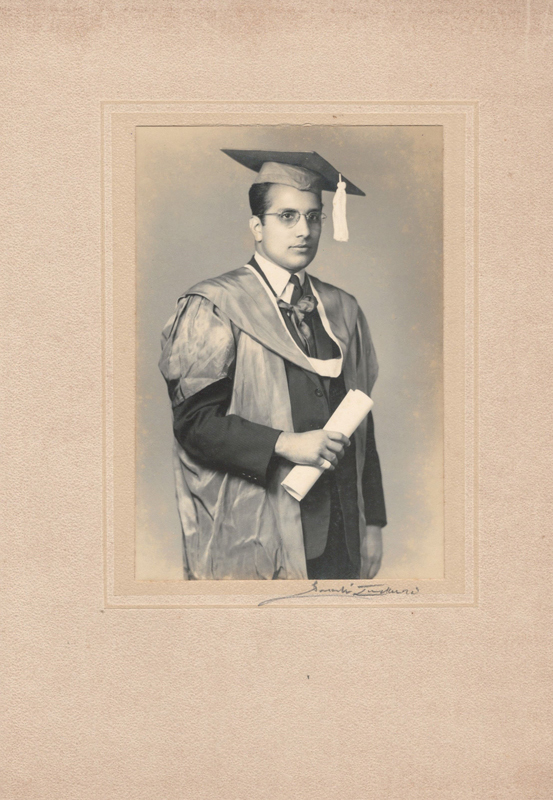
Medical graduation from King George's Medical College.

**Fig. 3:**
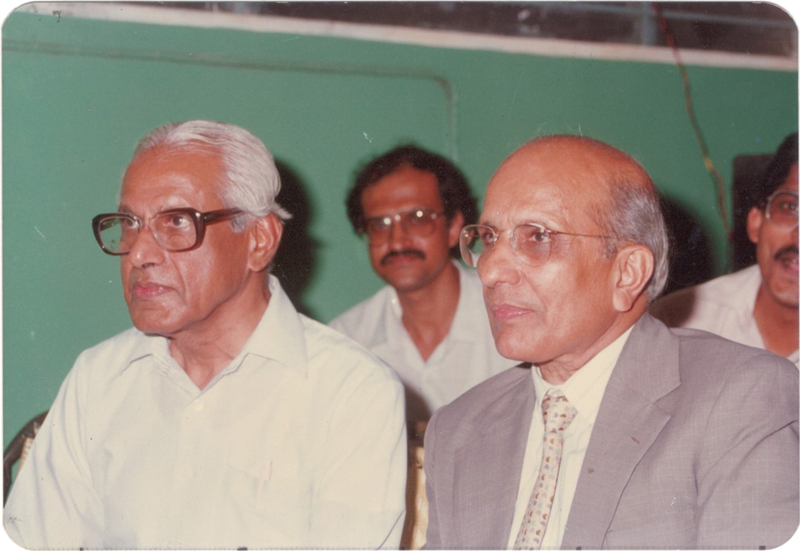
S.N. Sharma with his guru Prof. C. Balakrishnan.

**Fig. 4:**
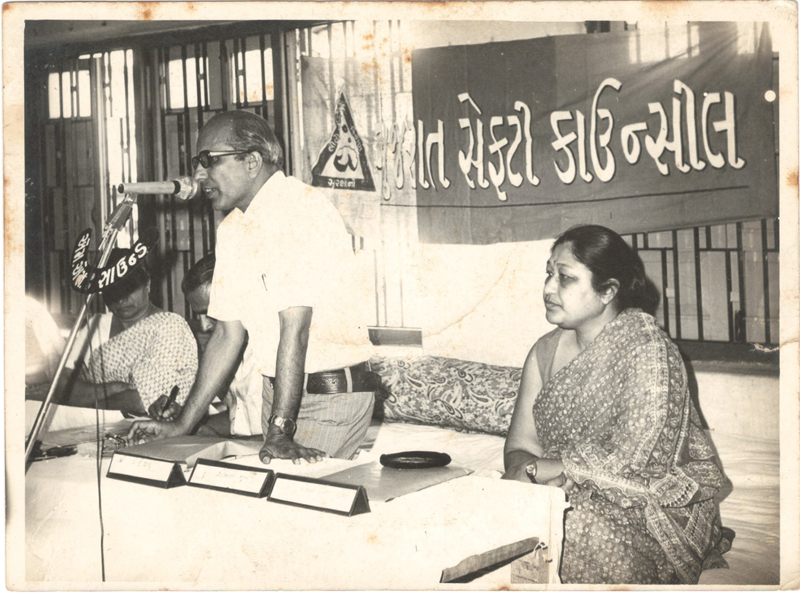
At Government Medical College, Baroda.

Dr. Surendra Nath Sharma completed MBBS in 1958 and MS in Surgery in 1962 from King George's Medical College, Lucknow ( Fig. 2 ). He credited Prof. C. Balakrishnan (CBK), the Father of Plastic Surgery in Modern India, for the success of his surgical career ( Fig. 3 ). The first MCh course in India had just started at Government Medical College, Nagpur, and he was the second batch student under Prof. CBK. Soon after completing his MCh in 1967, he worked briefly in PGI, Chandigarh, and then joined as an assistant professor of plastic surgery at Government Medical College & SSG Hospital, Baroda. He established an independent plastic surgery department (the first of its kind in Gujarat) and continued to serve as the professor and head till his retirement in 1992 and as an honorary professor till 2004 ( Fig. 4 ).

**Fig. 5:**
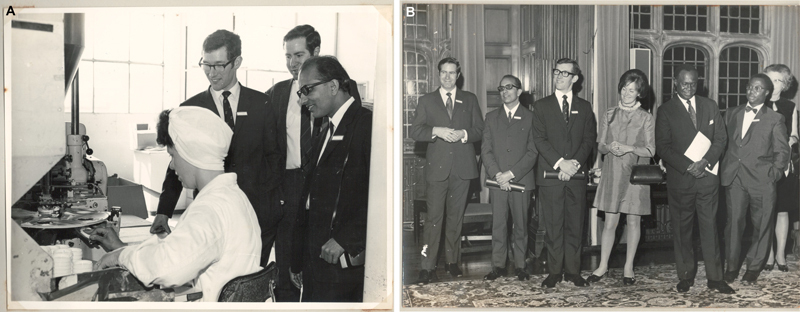
( **A** ) In the United Kingdom as part of the Smith and Nephew Plastic Surgery Travelling Fellowship team. ( **B** ) Visiting the Smith and Nephew research facility.

Dr. Sharma spent 1 year in the United Kingdom in 1970 as part of the Smith and Nephew Plastic Surgery Traveling Fellowship ( Fig. 5A ). This helped him bring back best practices to his department. He started the MCh Plastic Surgery training course in 1987 at Government Medical College under Maharaja Sayajirao University, the first center for the state of Gujarat.

He was a strong advocate of planning in plastic surgery and keeping solutions simple. He had a large series of cleft lip cases using Balakrishnan's triple wedge repair technique, and cleft palate and velopharyngeal incompetence repairs. Dr. Sharma promoted microsurgery by procuring an operating microscope in 1989, thus kickstarting limb replantation services in Gujarat.

Dr. Sharma regularly organized continuing medical education (CME) programs and workshops in the department and then under the Baroda Association of Plastic Surgeons (BAPS). His motivation ensured regular plastic surgery workshops being held in Baroda, and his camaraderie ensured esteemed national and international faculty conducting these. This put Baroda firmly on the plastic surgery academic map.

He was an MCh and DNB examiner for many universities and centers across the country. A keen advocate of plastic surgery, he encouraged many to take up the specialty and also referred many fresh graduates to major centers and individual surgeons overseas for further training in plastic, burns, maxillofacial, and cosmetic surgery.

## A Visionary President

**Fig. 6:**
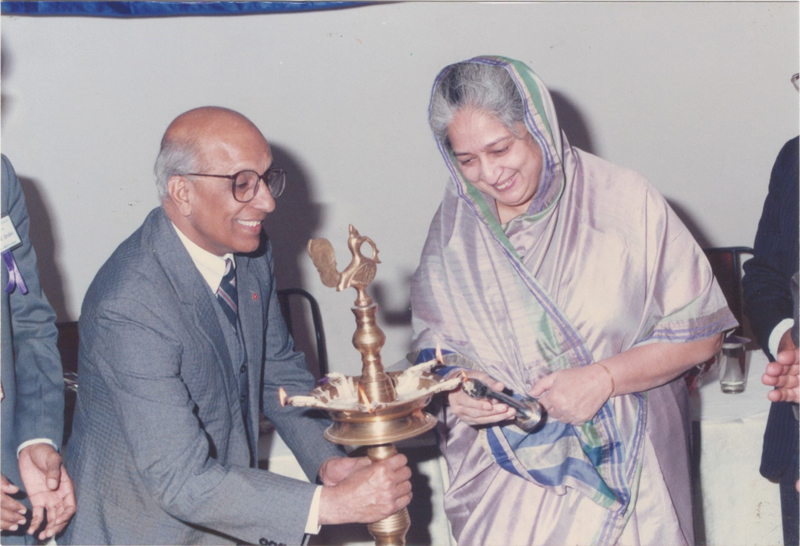
Prof. S.N. Sharma with Chancellor of MS University, Baroda, Mrinalini Devi Puar, lighting the inaugural lamp at Plastic Surgery Workshop.

**Fig. 7:**
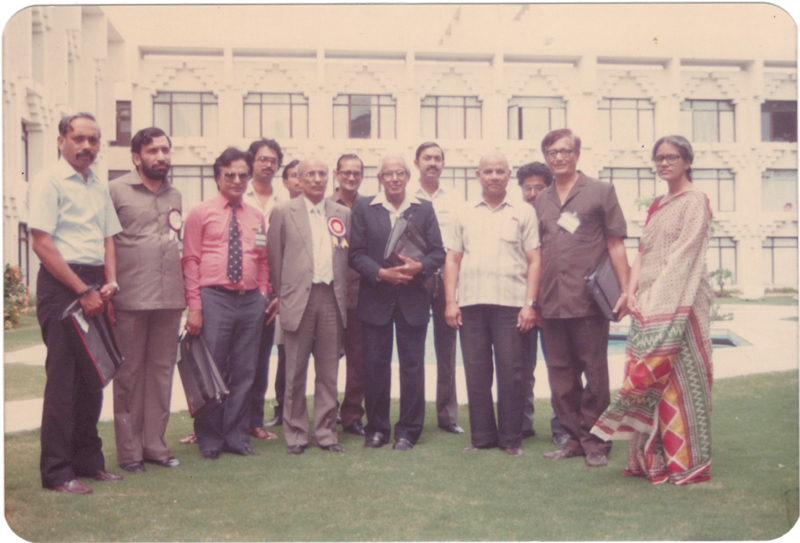
S.N. Sharma with C. Balakrishnan at APSICON 1989. Left to right: Dr. Karoon Aggarwal, Dr. Anil Chaddha, Dr. Ashok Govilla (pink shirt), Dr. Chiranjeev, Dr. Ramesh Sharma, Dr. S.N. Sharma, Prof. C. Balakrishnan, Dr. V. Bhattacharya, and others.

**Fig. 8:**
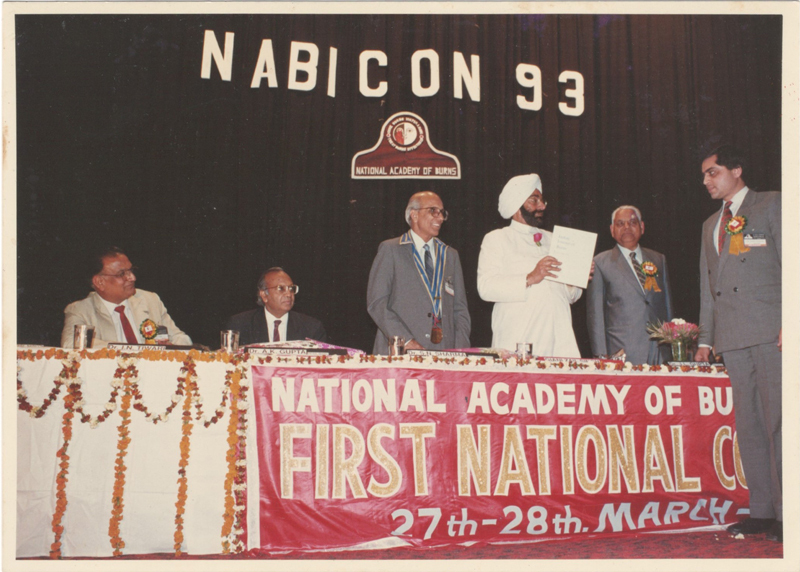
As founder president of National Academy of Burns-India (NABI), S.N. Sharma seen with Dr. J.L. Gupta and his excellency Giani Zail Singh, President of India, inaugurating the First National Conference of NABI in New Delhi, 1993.

Prof. Sharma was the president of APSI from 1988 to 1989 ( Fig. 6 ). He organized the first-ever operative workshop at APSICON 1989 at Baroda ( Fig. 7 ). It was also the First Aesthetic Surgery Workshop of APSI. He invited Dr. S. “Ari” Arumugam, Dr. Kulwant Singh Bhangoo, Dr. Sanjit Basak, and Dr. Vimla Rajan as international faculty ( Fig. 8 ). Many of the current senior generation plastic surgeons credit that workshop to be the inspiration and motivation to take up and master aesthetic surgery.

Prof. Sharma established and ran the APSI VIDEO lending library. He reached out to the doyens of the day in plastic surgery, both in India and overseas, requesting them to contribute videos of their pathbreaking/specialized work for the APSI video library to benefit its members and resident doctors. He later converted them to CDs as digital technology gained popularity. His residents were the key workers in the smooth functioning of this unique library. With the advent of online content, the library was discontinued.

Dr. Sharma delivered the Gilles Oration in 1991 on “The pursuit for perfection: triple wedge repair for cleft of the lip.”

At the behest of Prof. Mukunda Reddy, the S.N. Sharma Endowment Lecture was instituted in 2011 to be delivered at every APSICON by a distinguished expert in one of the fields of plastic surgery. This is supported by his family and students.

**Fig. 9:**
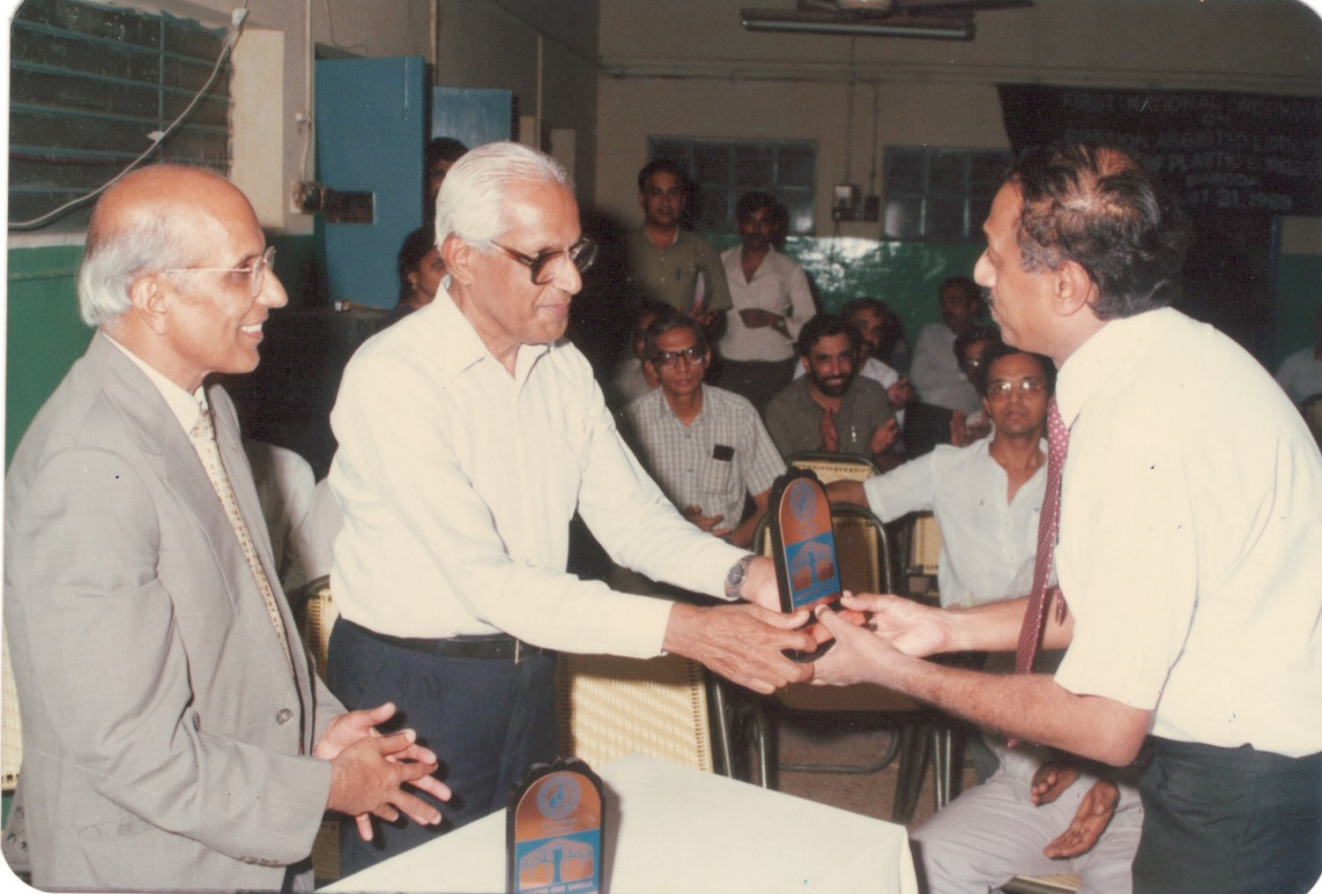
Prof. CBK felicitating Dr Ari Arumugam at the APSICON '89 Operative Workshop in Aesthetic Surgery.

Dr. S.N. Sharma was a keen advocate of burns prevention. He was the founder president of the National Academy of Burns-India (NABI; Fig. 9 ). He received the NABI Outstanding Service Award in 2001.

## A Luminary: Personal Life Style

A contented man, he lived by his ethics and values, often at the peril of his family members at home and staff at work. No situation flustered him, and I never saw him vent his frustration or voice self-pity.

His morning started with a run (5 a.m.) or a game of badminton. He would be ready and at the breakfast table sharp at 7.50 a.m. A nonfussy eater, he would leave home at 8.20 a.m. to be at the bedside of the first patient for ward rounds to start at sharp 8.30 a.m. Twice a week, his rounds involved visits to the patient toilets; thus, his kafila included the sanitary team too. The plastic surgery department stood out for its cleanliness, sterility protocols, and record keeping. He subscribed to journals, and his evenings were spent with these in one hand and coffee in the other.

A stickler for rules, he always advocated the right path even if it meant personal hardships. Also, he had only one set of rules, so be it a known person, an unknown person, or a family member, no favors were taken or given. Thus, there was no ambiguity around him. A strict disciplinarian, he led by example and expected tasks allocated and duties given to be completed diligently. I realize that he had created an environment that pushed everyone around him to give their best but never to break them.

He loved photography. His entry won the first prize in the railways' photography competition and his prize-winning photograph was featured on the cover of their national magazine in 1965. He had a vast collection of landscape, people, and portraits. However, as expected, his largest archive was of patient photographs.

He was a keen learner and always ready to sign up for anything new. A true believer in structured learning, he joined a 2-year computer course in 1992 at the age of 58 years, so as “to learn it properly.” On my return from Australia, after seeing me playing golf, he decided to take up the sport at the grand age of 70 years. An avid runner, he was thrilled when the first Delhi International Half Marathon was announced. He participated in the initial four Delhi and Mumbai marathons in his age category.

## Hobbies

*Traveling:* He loved the Himalayas. A 2-week sojourn there each year was a given. He enjoyed traveling and had visited each corner of India and more than 20 countries.

*Photography:* As probably with most plastic surgeons of that era, he had a huge cache of Kodak Ektachrome slides, capturing varied facets of life.

**Fig. 10:**
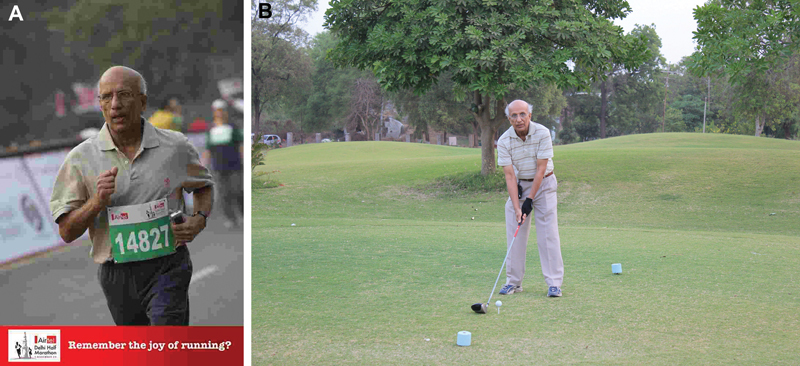
( **A** ) In action at the Airtel Delhi Half Marathon. ( **B** ) S.N. Sharma on the Gaekwad Baroda Golf Course.

*Sports:* Badminton, distance running, golf (late bloomer; Fig. 10 ).

## Family Man

Although he enjoyed a good life, he was never very possessive materialistically. Apart from his camera, he just prized his family, students, and friends. He had a high regard for knowledge and scientific achievement, and was never in awe of big popular names. In fact, he once famously refused to photograph a famous Bollywood star during a family vacation in Pahalgam to much social head shaking, commenting that there were enough magazines carrying those, so he would rather save film for more relevant subjects.

**Fig. 11:**
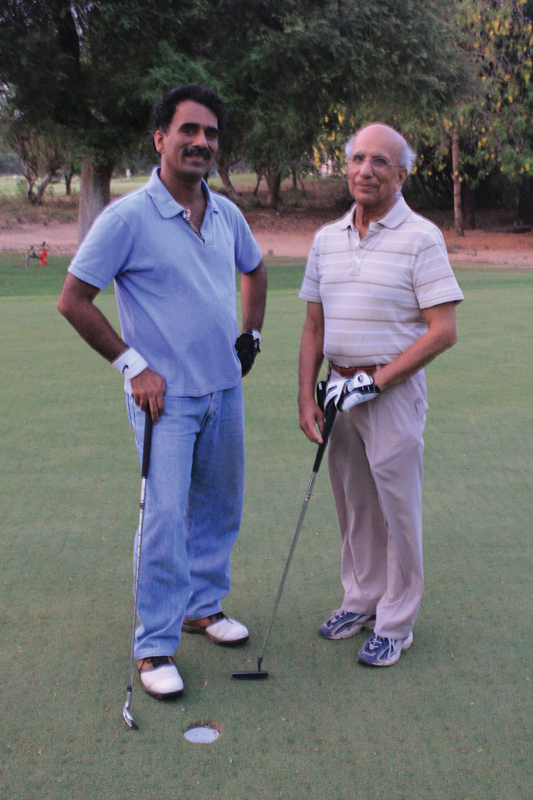
Father and son: S.N. Sharma and Sandeep Sharma (President IAAPS 2021–2022). His younger son, Prateek Sharma, is Prof. of GI, Kansas University, United States (President International Society for Diseases of the Esophagus [ISDE], 2018–2020, Current President of American Society of Gastrointestinal Endoscopy [ASGE], 2024–2026).

Lessons learnt (and missed) from his life ( Fig. 11 ):

- To create the right habits early on.
- Self-discipline.
- Constantly strive for physical fitness.
- Be your best, present your best, do your best—always.
- Integrity and honesty.
- High professional standards.
- Family man.

## Close Friends

In the days when communication was primarily through letters or personal visits, his closest friends were four of his batchmates from college, but he enjoyed the camaraderie with many plastic surgeons from around the country. Dr. C.P. Sawhney, Dr. J.L. Shrivastava, Dr. Ramesh Chandra, Dr. Ramakrishna Nair, Dr. Mathangi Ramakrishnan, Dr. S. Baliarsing, Dr. Anil Chaddha, and Dr. S.P. Bajaj were especially close. They and many more were regular visitors who, when in Baroda, invariably stayed with us. He was enthusiastically supported by my mother, Dr. Nirmal Sharma, a busy and locally popular gynecologist in her own right. Together, they diligently attended all APSICONs, which was the highlight of their annual social and academic calendar.

## Tributes

### Dr. Yogesh Bhatt, Former Professor and Head, Department of Plastic Surgery, Medical College, Baroda

Prof. S.N. Sharma sir's love for the branch was visible in all the hard work he put in and his lifelong efforts to help bring a good name. He set up a plastic surgery department at the Government Medical College, Vadodara, a pioneering feat in itself. He has the credit of starting the MCh program when no one else could have dreamt of it. It was not easy, but he fought against all odds to keep it running successfully.

He was particular about record keeping in the department. His meticulous data recording and analysis system was so perfect that his students are following it to this day due to its relevance even today.

The students he trained are well known today thanks to his perseverance, efforts, and relentless hard work.

His punctuality, dedication, and simplicity are some of the qualities we still remember. He was a strict taskmaster but a soft and lovingly fatherly figure at heart. The department and his students will always remember him as the father of plastic surgery in Gujarat.

Undoubtedly, he was named the president of APSI as early as 1989. He then successfully organized APSICON at Vadodara, a happily remembered and cherished event. The conference was one of its kind, for it included an operative workshop for the first time in India.

### Dr. Hiren Bhatt, Baroda

Prof. S.N. Sharma was a strict disciplinarian and was very punctual in his daily routine. He was well versed with all types of plastic surgery, but his main interest was in cleft surgeries, faciomaxillary injuries, and postburn reconstruction. He also encouraged his students to take up new subspecialities of plastic surgery. To overcome the handicap of training in aesthetic surgeries, he used his contacts with senior plastic surgeons both in India and overseas and organized annual aesthetic surgery workshops in the department of plastic surgery. The department soon became a very comprehensive department of plastic surgery, doing microvascular surgeries and welcoming aesthetic surgeries.

### Dr. Balwant Arora, Plastic Surgeon, Virginia, United States

I do not have enough words to describe sir's contributions to my life.

During my surgery residency, I realized that in plastic surgery, I can operate from head to toe, keeping in mind cosmesis. I can also continue to monitor the results of what I have done without too much of investigations. Hence, plastic surgery as a subspecialty was a natural choice.

Plastic surgery residency under sir was the best phase of medical education for me. With Sharma sir at the top, Hiren Bhatt being the batch mate, Vimal Amin and Hemant Saraiya as seniors, Atulbhai Shah as Clinical Assistant above them, and Dr. Ravi Tah joining as Assistant Professor, the plastic surgery family was complete. Behind the strict discipline of sir, there was soft and tender love as a father figure for all of us. The APSI meeting organized under his leadership had a personalized touch; nothing was left to chance and is still remembered today.

The Workshop on Liposuction and Rhinoplasty and the CME programs were educationally fulfilling and memorable. Meeting sir's teacher, Prof. Balakrishnan, was heartwarming. He allowed his triple wedge repair technique for cleft lip to be critically evaluated by letting me do a thesis on it during residency.

His punctuality was phenomenal. His insistence on paper work in those days was unparalleled even by today's standards in a highly litigative society.

His data collection, documentation, and preservation in that manual era were no less precise—perfect—than in the current era of digitalization. It helped me a lot getting into residency in surgery and then in plastic surgery here in the United States. Today, I am a board-certified plastic surgeon with a private practice in the United States and probably the only one who also holds an MCh plastic surgery degree from India.

His antiseptic and aseptic practices and, theater disciplines, and actual rigid adherence are still very much contextual even today; nothing new has come.

He inaugurated my hospital in Anand in 1991. He also visited me in the United States and I met him at almost every trip to India.

We, the residents, were equally cared for by Madam (Dr. Mrs. S.N. Sharma), and the brotherly love of Sandeep and Prateek always made us feel like a part of sir's family.

If I had to do it all over again, I would not change anything.

### Dr. Surjit Bhattacharya, Plastic Surgeon, Lucknow, Former President APSI and Editor *IJPS*

Prof. S.N. Sharma was an asset to the APSI. Besides serving in the executive council, he rose to the office of the president of the association in 1988–1989. The annual conference in Baroda in 1989 was way ahead of time, and the entire workshop was dedicated to aesthetic surgery. Even as a past president, he was very active, and on more than one occasion, he served as a returning officer in our association elections. His friendly attitude toward one and all and his neutrality made him a popular choice for conducting our elections. He was also the organizer of the association's video library, and in the pre-Internet era, these operating video cassettes were very popular among trainees. He was also an active member of the Association of Surgeons of India and participated actively in its state and national conferences. He was a key figure in popularizing our specialty in Baroda and Gujarat.

